# Addressing Clean Label Trends in Commercial Meat Processing: Strategies, Challenges and Insights from Consumer Perspectives

**DOI:** 10.3390/foods12102062

**Published:** 2023-05-20

**Authors:** Elena S. Inguglia, Zuo Song, Joseph P. Kerry, Maurice G. O’Sullivan, Ruth M. Hamill

**Affiliations:** 1 Teagasc Food Research Centre, D15 DY05 Dublin, Ireland; 2 School of Food and Nutritional Sciences, University College Cork, T12 E138 Cork, Ireland

**Keywords:** traditional processed meats, plant-based, hybrid products, functional ingredients, hydrocolloids, reformulation, food systems

## Abstract

Background: The concept of a clean label is difficult to define, even in common language, as the interpretation of what a “clean” food is differs from one person to another and from one organisation to another. The lack of a unique definition and regulations of what the term “clean” means, along with the growing consumer demand for more “natural” and healthier foods, is posing new challenges for manufacturers and ingredient producers. The meat industry, in particular, has been affected by this new movement owing to negative attitudes and feelings consumers associate with consuming processed meat products. Scope and approach: The review scope is to describe attributes and associations around the “clean” label term by analysing the most recent ingredients, additives and processing methods currently available for meat manufacturers. Their application in meat, plant-based alternatives and hybrid meat/plant products, current limitations and challenges presented in consumer perception, safety and potential impacts on product quality are also presented. Key findings and conclusions: The availability of a growing number of “clean” label ingredients provides a new suite of approaches that are available for application by meat processors to help overcome some of the negative connotations associated with processed meat products and also support plant-based meat alternatives and hybrids.

## 1. Introduction

Consumer demands for food products are always evolving and changing to reflect trends and new societal interests. Increasing awareness of artificial ingredients and a growing interest in more natural and sustainably produced food and food-derived ingredients underpin the recent trends that demonstrate consumers are seeking less processed foodstuffs, or what they perceive as less processed. A renewed interest in healthier and more natural versions of traditional meat products is a major component of this trend. Recently, consumer perceptions of meat and meat products has moved from seeing meat as a positive, fundamental part of the diet, a good source of minerals, vitamins and high quality protein to a more negative view, where the consumption of red meat and processed meat products has been associated with an increased risk of chronic diseases such as obesity, cancer and risk of stroke [1,2]. The reasoning behind this change in attitude towards processed meat products is understandable and logical in some cases, while questionable in others.

However, even against this backdrop, the global meat sector was valued at USD 897 billion in 2021, and the increase is forecast to USD 1354 billion by 2027 [3]. The volume of the fresh meat segment is expected to increase by 7.1% in 2024, and is expected to touch 136.20 billion kg by 2027 [4]. In particular, the demand for animal-based meat products is projected to reach additional 200 million tons per year by 2050 [5]. While the above figures present a positive market picture in relation to processed meats continued popularity, there is a strong negative view surrounding what is perceived to be an excessive manipulation of food products, a lack of transparency around practice and product labelling, and a vagueness surrounding the “naturalness” associated with the use of processes and ingredients employed in the manufacture of processed meat products [1,6]. Interestingly, the meat industry has never felt the need to inform or educate consumers as to why processed meat products are required to be manufactured or ever presented the consequences of not producing these products following the primary processing of meat-producing animals for fresh meat supply. Consequently, we have arrived at a situation where consumers are now driving the demand for product format and the industry responding with new ingredients and formulations to meet this requirement. The challenge is that there are no available regulations nor formal definition of “less processed”, “natural” or “clean label”.

In Europe, “natural” essentially means that the food is “comprised of natural ingredients, e.g., ingredients produced by nature, not the work of man or interfered with by man” [7]. In the US, the Food and Drug Administration (FDA), has yet to develop a definition for the use of the term natural; the United States Department of Agriculture (USDA) states that meat and poultry can be labelled as natural when it contains “no artificial ingredient or added colour and is only minimally processed” which refers to traditional processes such as smoking, roasting, freezing, drying, and fermenting; additionally, label claims such as “no artificial ingredients” must also be included to support the “natural” claim. Thus, meat products containing naturally fermented vinegars are legally allowed to carry a “natural” claim, while foods that contain nitrites are not allowed to be labelled as such. In response to these new requirements, the meat manufacturing sector is therefore refocusing and embracing innovative ingredients and novel processing technologies to meet consumer desires for healthfulness and naturalness, highlighted as the driving force behind the “clean label” trend.

### 1.1. Defining Clean Label

“Clean label” is a concept that extends beyond meat and across all food processing categories. It intends to remove or minimize negatively artificial ingredients in the product list or replace them with natural ingredients [8]. The concept of a “clean label” is difficult to define, even in common speech, as what a consumer considers to be a “clean label” food differs from one person to another. Consequently, what is precisely included or excluded in “clean label” products vary from one industry to another; moreover, interpretation of what a is considered a “clean label” product can be different in the attempted interpretation by legislators, retailers, processors and consumers. In general, “clean labelling” can be understood in three ways: (i) as (re)formulation to remove additives, flavours, preservatives, stabilizers, thickeners, and other ingredients (Low/no salt, low-fat claims) to create a more “natural” product with a simpler list of ingredients with little, or no, processing; (ii) in terms of consumer perception of how “natural” a product is, based on their reading of the product packaging and ingredients list; and (iii) the use of ingredients that are naturally derived, physically derived by processing natural ingredients but not chemically derived through processing, and based on ingredients that consumers could buy themselves through retailing outlets. According to the Institute for Food Technologists, for a product to be classified as clean “label”, it needs to have as few ingredients as possible, an easy-to-recognise name and no artificial flavours or synthetic chemicals that can be perceived as “unhealthy” and “unfamiliar” by the consumers [9].

The lack of a unique definition and set regulations around the exact meaning of what the terms “clean” and “natural” means, along with the growing consumer demand, is posing new challenges for manufacturers and ingredient producers. “Clean label” is also inextricably linked to the search for presumed healthier products. In this context, comprehensive reviews looking at the strategies and ingredients to produce meat products with less fat [10,11], less sodium [12,13,14] and absence/replacement of nitrate and phosphate [15,16,17] have been recently published, highlighting the vast body of research and funding that has gone into this sector to identify valuable options and alternatives.

### 1.2. Role of Consumers

The rise of the “clean label” trend can be attributed in the first place to consumer themselves. More than ever, consumers are concerned about their personal health and well-being and are willing to pay a premium price for products and switch from their usual brands to those providing more informative product labels [18]. As highlighted by Aschemann-Witzel, Varela et al. [19], this trend also triggers consumers to turn to products, such as certified organic food, allergen-free and related claims driven by modern health concerns [20], negative associations with chemicals [21], as well as scepticism about functional food developments [22] and unknown ingredients [23]. In response, there have been worldwide investments to explore novel ingredients and to gain insights on consumers’ perceptions of “clean label”. It has been estimated that the global “clean label” ingredients market will be worth $51.14 billion by 2024 [24]. Several studies have examined the factors that influence people’s perception of naturalness [23,25,26,27,28,29]; this perception is different based on age, gender and location, with a tendency for younger generations (≤18 years old) to be more critical of the ingredients used [29]. In general, the three main categories used by consumers to identify what is “natural” are linked to food origin, food processing (technology and ingredients) and, lastly, properties of the final product [28]. Interestingly, another study from Siegrist and Sütterlin [30] showed that perceived naturalness decreased, independently of whether the food was synthetic or natural, with the presence of three food additives displaying their respective E-numbers in products. However, in another scenario, mentioning potential health effects decreased the perceived naturalness of a plant-based natural food additive [30]. Interestingly, as observed by Cheung, Junghans et al. [31], only a few consumers pay attention to the ingredients list when evaluating the “naturalness” of the food product upon purchase; however, in the presence of package or labelling indicators, the attention to these features increases.

### 1.3. Opportunity for Processors

**Figure 1:**
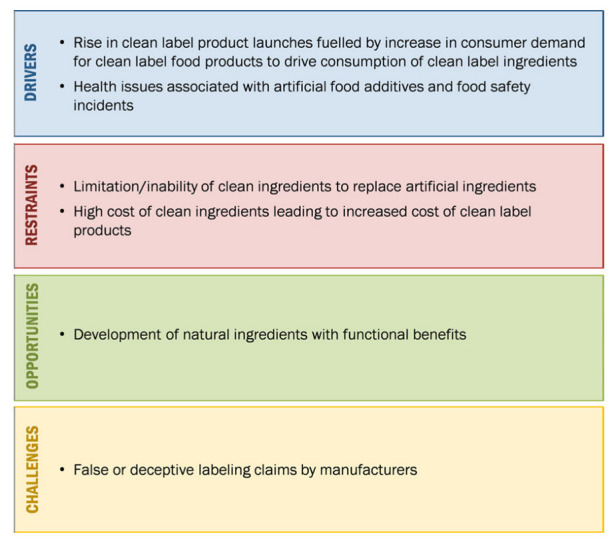
Market dynamics: Clean label ingredients Source: Modified image from MarketsandMarkets Analysis (Report Code FB4853).

In the face of demand from consumers, processors are presented with many challenges, but also opportunities for the innovation and creation of new products in the frame of clean label. Taste, texture, freshness and appearance are some of the major qualities that are influenced by the addition or removal of ingredients and additives in food products. Inclusion levels and interactions among ingredients have complex influence on product properties and must be considered when developing formulations with specific targets in mind. Based on the final conditions that need to be achieved, reformulations can be performed by (i) the partial or total replacement of a component, (ii) by the addition of extra components, (iii) by improving the component stability and bioavailability or (iv) by a combination of all of these measures [32]. Food ingredients and additives can be of various origins as well as functions. Generally, plant-derived ingredients have the advantage of being readily accepted by consumers, as by definition they are considered natural, whereas some food additives come from mineral sources. Those mineral-based additives that contain phosphorous compounds, have beneficial effects on juiciness and texture, helping to retain moisture in foods. Plant-based additives, such as seaweeds, are naturally rich in polysaccharides and minerals; based on the required product characteristics, they can be used in foods to modify and optimise texture and flavour. A great number of innovative food ingredients are obtained from crops and fruit waste (e.g., peel, rind, pulp) with observed antioxidant functions, while some other ingredients are of a microbiological nature, for example, probiotics, yeast and yeast extracts, mycelial mass, enzymes, etc., which can be added to food to improve digestive health, enhance flavouring/seasoning of savoury products, partially replace meat components and improve textural product properties, respectively. The availability of a growing number of “clean label” ingredients provides a new suite of approaches that are available for application by meat processors to innovate more clean label and natural processed meat products (Figure 1).

The purpose of this review is to describe attributes and associations around “clean label” and to analyse the most recent ingredients, additives and processing methods currently available for meat manufacture that can be used to meet or approach a clean label claim. Their uses, current limitations and challenges of use with respect to consumer perception, safety and potential effect on product quality are also discussed. Several important categories of clean label ingredients and additives with relevance to meat products will be discussed. Examples will illustrate applications in the reduction in, or possible elimination of, undesirable or unnatural food processing ingredients, such as fat, salt, nitrites, nitrates and phosphates, and for overcoming challenges with the inclusion of physiologically relevant levels of vitamins and antioxidants to formulate clean label meat products and, recently, the inclusion of a proportion of plant proteins in hybrid products, or fully plant-based meat analogues.

## 2. Clean Label Ingredients

### 2.1. Role of Hydrocolloids

Clean label ingredients come from a variety of sources, among which hydrocolloids are the most commonly used category in the food industry (see Table 1). The term “hydrocolloid” is used in a variety of industries to describe polymers that have gelling, thickening and stabilising functions, in addition to attributes of specific relevance to food formulation, i.e., valuable source of dietary fibres [33]. Many polysaccharides of vegetable origin are currently available on the market, such as starches (corn, wheat, maize, potato, tapioca, pea), celluloses (methylcellulose), gums (guar, alginate, pectin, locust bean), fibres (ß-glucan), chitin/chitosan- and xanthan-derived from microorganisms [34]. Application of “clean label” hydrocolloids in the meat processing and plant-based alternatives sectors (for examples, see Table 2 and Table 3), has been shown to improve properties such as water binding capacity, texture and emulsion stability due to their ability to thicken, gel and bind meat pieces together, thereby making them particularly suitable for the reduction of fat, salt and phosphate from meat products [35]. By retaining water, hydrocolloids can change the viscosity of the food product, influencing the release of aroma compounds as well as contributing to the overall mouth feel of the product [36]. Most hydrocolloids are labelled as food additives but despite their “natural” origin, they are not always seen as “clean label” by consumers [27]. As observed by Song and Schwarz [37] and Varela and Fiszman [27], additives that have hard-to-pronounce names are perceived by consumers as more harmful than compounds with simpler names; pectin for example, has the best public image, while similar hydrocolloids such a carboxymethylcellulose and carrageenan have a negative or unfamiliar connotation, and there is a risk that consumers conscious of ingredient labels will refuse food products with such components. Additionally, consumers considered a food ingredients listing to be more acceptable and healthier when presented as having a natural source, even if modified so the terms “modified potato, tapioca or corn starch” are more acceptable than “modified starch”. This suggests that when the origin of food ingredients are known, consumers perceive the ingredients as being more natural and are more accepting of the ingredients in question.

#### 2.1.1. Applications to Fat Reduction and Replacement

Fat content in processed meat products differs broadly based on the type of meat (e.g., chicken, turkey, pork, lamb or beef) and the type of product (e.g., deli meats, sausages, patties, nuggets, etc.) with values that range from <5% up to >30% [10]. Fat has a major effect on texture, juiciness, mouth feeling and flavour of the meat products [38]. Functionally, both non-polar and polar myosin components involved in connecting fat cells to the water phase are ultimately responsible for emulsification and water-holding capacity [39]. Fat reduction, without reformulation, can lead to undesirable texture (rubbery, dry texture, hardness, decreased tenderness and juiciness), unpleasant flavour and undesired sensory properties [40]. Hydrocolloids may partially replace fat during the manufacture of low-fat meat products. Studies have shown that the use of inulin and pectin as fat substitutes increased yield and moisture content in low-fat sausages [41,42]. In low-fat chicken patties, the pre-emulsion replacement of pork loin fat with wheat sprout and collagen by up to 10% was shown to improve the quality characteristics of the product, while the use of hydrated wheat fibre was shown to replace up to 44% meat and fat in burgers [43,44]. Similarly, significantly lower cooking losses and improved water binding capacity were determined by adding 2%(*w*/*w*) powdered fibre (inulin, cellulose, carboxymethyl cellulose, chitosan and pectin) to a fat-reduced meat model system, while the impact on textural properties was observed to be dependent on the specific dietary fibre used [45]. The incorporation of “clean label” ingredients, such as grape seed oil and rice bran fibre, successfully reduced animal fat content in meat emulsions systems by 10% [46], while the application of grape seed oil (10%) formulated with the addition of gelatine and alginate had a significant effect on the physicochemical properties of meat emulsions [47]. Hoary basil mucilage is a novel hydrocolloid that has been recently attracting interest for its potential usage in food products. Its role as a pork back-fat replacer, using up to 80% in chicken meat batter with 2% salt, was evaluated. Results showed that sensory perception of this ingredient was no different from the control used in experimental trials, and consequently, the chicken meat model was accepted by consumers [48]. Chia seeds mucilage is also a relatively new ingredient in the hydrocolloid area, as it can be used as a functional ingredient, owing to its ability to form gels, in emulsified meat model systems. A 50% substitution of pork back-fat with chia mucilage gels in meat model systems was tested at three different concentrations of 15%, 20%, and 25% (Table 3), applied at two levels (2.5 and 5.0%) and the results showed that chia substitution for saturated fat in emulsified meat products improved technological characteristics and, ultimately, health claims [49].

Regardless of the functional properties or clean labelling of low-fat meat products, if they are scored not acceptable in terms of palatability or appearance, the product will not be well received by consumers. Depending on the different meat types and products to be produced, reducing fat to between 5–10% can often result in reduced flavour, reduced cook yields, increased dry and hard textures, etc. Therefore, to compensate for this, the moisture to fat ratio must be increased, therefore implying adjustment in spices and other flavouring levels. Additionally, the use of fat substitutes can also have negative effects on products, and reformulated products may have reduced particle binding, darker product colour, lack of meaty flavour and a shorter shelf-life [41]. Moreover, replacements should contribute to a minimum of calories in the product and should not negatively impact on organoleptic qualities.

#### 2.1.2. Applications in Plant-Based Meat Alternatives and Hybrid Meat Products

Along with the concerns around health and clean label drivers, processed meat consumption is facing challenges to reduce carbon footprint and increase sustainability and welfare around animal production [50,51,52,53]. The global demand for high-quality protein continues to increase, thus interest in alternative protein sources has grown rapidly over the past decade [52,53,54]. The protein sector is diversifying through increased provision of proteins from plants, fungi, edible insects, animal stem cells, precision fermentation, and microbial cells [54,55]. Meat alternatives, also named meat substitutes, meat analogues among other terms [56,57,58,59], are meat-like foods made from non-meat ingredients. Plant-based meat alternatives are food products that are manufactured from (generally but not always) textured proteins extracted from plants [60,61]. They have rapidly gained popularity and are currently the most preferred type of meat alternative [61]. The global plant-based meat market, which was valued approximately USD 11.92 billion in 2018, is predicted to reach around USD 21.23 billion by 2025 according to the report from Zion Market Research [62].

Although plant-based meat alternatives offer numerous advantages, their market share remains relatively low, representing 1% relative to the total meat market [61]. The main challenges to stimulate expansion of market share for plant-based meat alternatives have related to the consumer attitude, such as unfamiliarity of meat alternatives [61], an aspect which is likely reducing as they become more to the mainstream but, in particular, is due to their inferior textural and sensorial properties compared with 100% meat-based products [63]. To enhance consumer acceptance of plant-based meat alternatives, a combination of technical innovations and new product formulations is a key area of research seeking to deliver a meat-like texture while maintaining similar nutritional properties to animal meat. As there are inherent differences between muscle tissue and plant-based ingredients, formulation is playing an important role [64]. A typical formulation of plant-based meat alternative contains protein ingredients, water, flavourings, oil or fat, binding agents, and colouring agents. However, the lack of clean label in the formulation is another common challenge for plant-based meat alternatives [64]. In order to achieve meat-similar sensory attributes and nutritional profiles, a large number of additives include preservatives, stabilizers, and colorants are incorporated in the alternative products formulations [64]. The extensive refining process used to create these additives has led to criticism that plant-based meat alternatives are artificial [65]. Such plant-based products are not well perceived by those consumers who prefer more natural ingredients [65]. Clean label hydrocolloids possess gelling, thickening, emulsifying, and stabilizing properties, which result from their ability to interact with water, proteins, starch, and other components present in food products [63,66]. Some hydrocolloids have been incorporated to address the challenges associated with replicating meat-like textures with plant protein (Table 2), and these have high relevance in the plant-based space, in particular carboxymethyl cellulose (CMC), which contributes desired textured attributes to plant-based meat alternatives [63,65]. CMC is a hydrocolloid that is soluble in water and carries a negative charge due to its carboxyl group, allowing it to form robust networks or complexes by interacting with the positively charged protein domains [67]. CMC helps transform the plant-based meat alternatives into fibrous materials, furthermore, the research suggested it would be interesting to evaluate whether incorporating a combination of CMC and other hydrocolloids such as xanthan (X) could improve the textural and sensorial attributes of plant-based meat alternatives [63]. As shown in Table 2, a range of other hydrocolloids have also already been tested in plant-based meat alternatives. For example, the effects of using hydrocolloids, including κ-carrageenan, konjac mannan and xanthan gum, on physical and sensory characteristics of meat-free sausages was investigated [68]. Up to 0.6% Konjac mannan and κ-carrageenan resulted in an enhanced overall acceptability of the plant-based sausages, significant improvement in water-holding capacity, texture, and reduction of cooking loss. Thus, these hydrocolloids possess the ability to create a robust network within the sausage matrix. Additionally, it is plausible that these hydrocolloids can form a stronger network with other sausage components, such as soy proteins and starch, to promote the structural stability and integrity of the sausages [68]. Similarly, Palsnisamy et al. [69] investigated the effect of adding 0.75–3% of iota carrageenan on the physical properties, texture, sensory parameters and microstructure of soya meat analogues produced by high-moisture extrusion processing. The results showed that increasing iota carrageenan inclusion levels led to a more compact network in the meat analogues, supporting the changes found in the texture, cooking yield, and expressible moisture. Achieving superior physical, textural, and sensorial properties in soy-based meat analogues can be accomplished by formulating them with 1.5% iota carrageenan.

Although consumers are often aware of the issues associated with meat consumption and the benefits associated with plant-based diets, the idea of reducing personal meat consumption is met with resistance [70]. For individuals looking to adopt a flexitarian or semi-vegetarian diet, incorporating hybrid meat products can be a useful approach. These products replace a significant portion of meat with alternative proteins, allowing consumers to increase their intake of plant-based proteins while still enjoying the taste and texture of meat-based products; however, several studies have found that incorporating plant-based proteins into meat products can lead to a less robust network formation and a softer texture [71,72,73]. To address this issue, hydrocolloids have been utilized in the formulation of hybrid meat products. For example, 0.9% CMC was incorporated to make hybrid chicken sausages [74]. Potato starch was incorporated into the formulation of the hybrid meatballs at a rate of 2% [75]. In addition, the use of functional ingredients (such as hydrocolloids and proteins) was suggested to be incorporated into the formulation to improve the structure and physical stability of hybrid meat products [76].

**Table 2:** Applications of hydrocolloids in plant-based meat alternatives.

Hydrocolloid	Applications	Approach	Findings	Reference
Konjac glucomannan	Fermented soybean patty	Addition 0, 2, 4 and 6% *w*/*w* konjac glucomannan in fermented soybean patty	Konjac improved overall the textural and, potentially, eating quality and moisture retention of fermented soybean patty. The addition of konjac strengthened the protein–protein network. The meat alternatives with the incorporation of 6% konjac was the most cohesive among all samples, which was closer to the cohesiveness of the meat patty.	[77]
κ-carrageenan, konjac mannan, xanthan gum	Soy-protein-isolate sausage	Supplementation of κ-carrageenan, konjac mannan and xanthan gum at concentrations of 0.3%, 0.6%, 1.0%, and 1.5% was applied to a soy-protein-isolate sausage	The application of 0.3–0.6% kappa-carrageenan or 0.6% konjac mannan yielded the most favourable acceptability scores.	[68]
Iota-carrageenan	Soy protein meat analogues produced by high-moisture extrusion processing	Addition of iota-carrageenan at 0.75%, 1.5%, 2.25%, 3% to soy protein meat analogues	1.5% iota-carrageenan was the optimal level for acceptance of texture.	[69]
Xanthan (X), iota-carrageenan (CA), sodium alginate (SA), guar gum (GG), carboxymethyl cellulose (CMC), low-acyl gellan gum (GZ), low-methylated pectin (P), locust bean gum (LBG)	Pea protein isolate-wheat gluten (PPI-WG) products produced by high-temperature shear cell processing	Addition of different hydrocolloids (X, CA, SA, GG, CMC, GZ, P, LBG) at 1%, 2%, 3% to the PPI-WG mixture	The addition of X at 2 and 3% to PPI-WG improved browning, textural and water-holding properties in the production of fibrous products.	[63]
Guar gum (G), κ-carrageenan (C), xanthan gum (X), hydroxypropyl starch (HPS), cross-linked tapioca starch (CLS)	Soy-protein-isolate-based meat analogue produced by twin-screw extruder	Gluten substituted with G, C, X, HPS, or CLS at concentrations ranging from 1–7% (*w*/*w*) in the control recipe	The mixtures of 6% C + 1% X and 6% G + 1% X can be used for substituting gluten in a soy-protein-based meat analogue.	[78]
Carrageenan (CA), sodium alginate (SA), wheat starch (WS)	Peanut protein powder (PPP) meat alternative processed by high-moisture extrusion	Addition of CA/SA at 0.05%, 0.1%, 0.5%, and 1% to the PPP. Addition of WS at 2%, 4%, 6%, and 8% to the PPP	Overall, 0.1% CA could improve the tensile resistant force, and 0.1% SA could improve the fibrous degree. Increasing the WS content (0–8%) resulted in a lower fibrous degree and a significant reduction in both hardness and chewiness.	[79]
Kappa- and iota-carrageenan (CG), sodium alginate (SA), glucomannan (GM)	Meat analogues produced by three-dimensional food printing	Insertion of hydrocolloid-based fibres prepared in 2.5–5% solutions with different formulations of CG, SA, and GM into the protein matrix	When subjected to heat treatment, the combination of 1.5% CG and 1.5% GM, as well as 2.5% CG and 1.5% GM, formed a highly robust and stable gel with an elastic strength similar to that of beef.	[80]

### 2.2. Clean Strategies for Salt and Phosphate Reduction or Replacement

The addition of salt (NaCl) in meat products has a number of important roles to fulfil. Its primary function is to solubilise myofibrillar proteins to generate functionality within processed meat systems, thereby producing properly structured and textured products. A secondary role is to reduce water activity in processed meats, thereby promoting its preservative effects. A tertiary role is, of course, to deliver a unique flavour enhancing profile in processed meat products. Salt reduction in food and meat products has been a priority for more than a decade, with a variety of strategies employed to address this need [12]. On the “clean label” spectrum of proposed strategies, ingredients that can promote the umami flavour, thereby increasing salivation, are often preferred in a formulation as they promote the overall recipe taste balance. The inclusion of 2.5% mushroom flour (Table 3) from *Agaricus bisporus* and *Pleurotus ostreatus*, for example, have been reported as feasible alternatives to reducing the salt content of beef patties by 50% while maintaining an acceptable sensory profile [81]. Seaweeds, such as sea spaghetti, wakame and nori, are rich sources of minerals, which can act as flavour enhancers and may replenish lost flavour in reduced salt and fat processed meat products [82]. Additionally, seaweed ingredients have received a lot of interest in recent years as functional ingredients since their addition to meat formulations can be a source of polysaccharides, thereby improving the structure and strength of reduced-fat products, providing bioactive substances, fibre and additional umami flavours for reduced sodium products [83]. A recent comprehensive review investigated a multitude of food applications employing seaweeds, concluding that a wide range of uses are possible in meat products using seaweed as a “clean label” ingredient [84]. Another well-known hydrocolloid used in meat products is konjac gel. A combination of sea spaghetti/konjac gel was used to produce low-fat (1.7%) and reduced-fat (10.5%) frankfurters employing 1% salt. This fat blend decreased cooking yield and emulsion stability while still maintaining an acceptable sensory score for low-salt frankfurters [85]. Other ingredients, such as native starches, are very effective in poultry products due to their light colour, clean flavour, and because they activate at lower cooking temperatures.

Phosphates are commonly used in processed meats to improve texture, promote water-holding capacity, increase ionic strength, and chelate divalent cations, which helps maintain and increase the optimal pH level. Salt is used primarily for flavour and preservation purposes and can increase water-holding capacity, [16]. To compensate for the negative effects of salt and phosphate reduction, other ingredients must be added to improve textural parameters and water-binding properties of meat products. Historically, “clean label” phosphate replacement has focused on the improving the water-binding capacity of the meat system in question. Ingredients such as citrus and vegetable-based fibre sources, for example, can bind to a lot of water without requiring heat and are becoming interesting options for phosphate replacement. For example, multiple functional ingredients can be derived from plums, including substances such as pectin and sorbitol, which are effective in moisture retention, with malic acid functioning as a flavour enhancer. Applications to poultry products have shown that the combination of plum powder and plum fibre marinade was found to have similar sensory and quality characteristics when compared to sodium tripolyphosphate in boneless and skinless chicken breast fillets [86]. In addition to phosphates, proteins from various sources, such as meat (collagen), dairy, and plants, can also be used to improve the yield of processed meat products. Isolated soy proteins and soy protein concentrates are the predominant products used in many processed meats due to their high solubility, clean flavour and economical cost [87]. Alternatively, unique ingredient combinations are now being used commercially to replace phosphates in meats that include fruits and vegetables, allowing the labelling recommendation as “natural flavourings”. Recently, chia mucilage powder at 2% has again been proven to be a feasible strategy in the substitution of 50% of the phosphate in low-fat Bologna sausages, owing to its techno-functional properties that mimic phosphate by binding proteins to water and increasing water-binding capacity naturally [49].

During meat processing, one of the most important step is the extraction of salt soluble proteins from meat with NaCl [88,89]. When reducing salt, increasing spices or acidity can help to improve flavour, but do not provide the same technical functionality that may be associated with reduced product quality, yield and texture in salt-reduced food matrices. The increased demand for natural meat and poultry products, especially as the term “natural” relates to avoiding ingredients with chemical names, has significantly reduced the number of ingredient options. For instance, potassium versions of existing sodium salts, which typically have equivalent functionality, are not acceptable under this scenario. The use of natural compounds to replace sodium-containing ingredients is positive to consumers, but for processors the issue is to have ingredients that can replace others in terms of functionality. Similarly, several phosphate replacers and substitutes, such as proteins, carrageenan, starches and other fibres, can address phosphate’s important water-binding function; however, they are not necessarily able to provide other associated functions, such as antioxidative potential, leading to the necessary addition of other ingredients to compensate for reduced function. An important element of safety concern surrounds reduction of salt content in processed meat products as previously described. Therefore, careful consideration must be given to shelf-life and stability studies prior to a product’s launch. Consequently, the application of novel minimal processing technologies can help improve the stability and preservation of novel formulations; however, their commercial applications are still limited [12]. Moreover, allergen concerns must be considered when novel binders, such as soya or milk proteins, are introduced in the meat system. In addition to the advantageous functions offered by salt and phosphates, they are relatively cheap to use compared with substitutes, therefore, the willingness of the consumer to pay more for a natural product should be carefully assessed.

### 2.3. Clean Strategies for Antioxidant and Antimicrobial Functionality

Lipid oxidation, microbial growth and enzymatic autolysis, including proteolysis and lipolysis, are the three main causes of spoilage in processed meat products. Lipids are particularly sensitive to the UV component of light, oxygen, storage temperature as well as processing methods. The lipid oxidation process, once commenced, can lead to the formation of other compounds causing changes in the colour, texture and flavour of the product [90]. One way to limit or inhibit oxidation is through the use of antioxidants, thereby improving the quality and shelf-life of products. Common synthetic antioxidant compounds typically include BHA (butylated hydroxyanisole), BHT (butylated hydroxytoluene), propyl gallate, and TBHQ (tert-butylhydroquinone) mostly used in the US market, or sulphur dioxide (sodium metabisulphite) used more in Europe, UK, Australia and New Zealand. Commercially available natural compounds belong to the family of phenolics, such as phenolic acids, tocopherol, and flavonoids. Phenolic compounds, such as carnosic acid from rosemary and catechins from green tea, prevents lipid oxidation by functioning either as free-radical scavengers or metal chelators, preventing the oxidative breakdown of meat pigments [91,92,93]. Rosemary and green tea extracts are proven ingredients for their positive impact on the appearance, taste and quality of meat and poultry products, with green tea extracts having only minor effects on final product flavour. This characteristic allows the manufacturer to use a blend of the two extracts, increasing the total quantity of natural antioxidant going into the product and minimizing the potential negative effects [94]. Seaweed extracts containing fucoidans, have been shown to enhance antioxidant activity of functional cooked meat products [95,96]. Acerola cherry extract has also shown to be a highly effective ingredient in meat and poultry and is extracted from a wild plant grown in tropical and subtropical regions. Acerola extract provides a high quantity of vitamin C. The ingredient has been shown to delay both lipid and myoglobin oxidation, thereby delaying the onset of colour loss and maintaining the desirable colour and quality of meat products. When used in combination with rosemary and green tea extracts, acerola is more effective at delaying early discoloration than either extract alone. Similarly, a blend of rosemary extract (0.5%) combined with buffered vinegar, rosemary extract (0.2%) and green tea has been shown to be effective in prolonging the shelf-life of sausages to the same extent as synthetic antioxidants, such as BHA and BHT used at 0.02% fat content [97]. Natural phenolic-rich berry extracts, including bearberry *(Arctostaphylos* sp.), blueberry (*Vaccinium* sp.), blackberry (*Rubus* sp.), blackcurrant (*Ribes nigrum*), cranberry (*Vaccinium* sp.), cloudberry (*Rubus chamaemorus*), strawberry (*Fragaria ananassa*), and grape (*Vitis* sp.), have been shown to possess a strong inhibitory effect on meat oxidation [32]. In addition to plant extracts, certain spices, fruits and vegetables are associated with preserving the colour of meat. Dried plum ingredients are an example; not only are they a source of antioxidants, but they can also contribute to a desirable red hue, therefore enhancing the colour and its stability in meat products [98]. Some of these plant extracts, generally those with high concentrations of polyphenols/flavonoids and antioxidants, have also been shown to be effective against specific pathogens. Common antimicrobials used in processed meats include sodium lactate, sodium diacetate, sodium propionate, potassium lactate, potassium acetate and vinegar. The increasing number of products requiring “clean” labelling has led to an increase in the use of vinegar as a “go to antimicrobial”. Vinegar derivatives are effective against microorganisms and are considered natural. Different versions of vinegar, such as liquid, dry and distilled, are also used more on “clean label” products since such materials are perceived as less processed [99,100,101]. Cranberry pomace is another example of an ingredient with significant antibacterial activity against *Escherichia coli*, *Salmonella ser*. Enteritidis, *Listeria monocytogenes*, and *Staphylococcus aureus* in minced pork [102]. Other natural antimicrobials used to control the growth of *Clostridium perfringens* in frankfurters and hams were blends of cultured sugar and vinegar and a blend of cherry, lemon, and vinegar powder [103]. Moreover, ingredient suppliers are continuing to customize blends for specific applications for both fresh and ready-to-eat (RTE) meat and poultry products to preserve colour and flavour, while providing protection against pathogens.

**Table 3:** Ingredients used in the development of clean-label meat products.

Ingredient	Inclusion Level	Meat Product	Impact on Product	Contribution	Reference
Inulin, pectin	15% inulin, 30% inulin, 7.5% inulin and 7.5% pectin, 15% inulin and 15% pectin	Frankfurter sausage	Fat can be replaced with inulin and pectin in frankfurter sausages. The addition of 15% inulin increased the sensory acceptance of the sausages.	Fat reduction/replacement	[42]
Wheat sprout	0%, 1%, and 2% buckwheat spout powder	Chicken patty	By incorporating 2% wheat sprout dietary fibre, the fat content in chicken patties can be reduced to 15% while maintaining the quality and sensory characteristics of patties containing 20% fat.	Fat reduction/replacement	[104]
Chia mucilage	15%, 20%, and 25% chia mucilage gels were applied in two levels (2.5% and 5%)	Emulsified meat model system	The addition of chia mucilage gel enhanced the stability of the meat emulsion. Formulations with 5% chia mucilage increased hardness, and decreased elasticity and cohesiveness values compared to the control with 20% fat.	Fat reduction/replacement	[49]
Inulin, cellulose, carboxymethyl cellulose (CMC), chitosan, pectin	2%	Pork meat model system	Fibre enrichment resulted in lower cooking loss and improved water-holding capacity. Chitosan impacted the heating-induced changes in water distribution. CMC exhibited a superior ability in mitigating the effect of heat-induced protein denaturation on water expulsion than the other fibre types.	Fat reduction/replacement	[45]
Carboxymethyl cellulose (CMC), microcrystalline cellulose (MCC)	0, 0.3, 0.5, 0.7, 1.0, 1.5 and 2 wt.%	Standard-fat Lyoner sausages	MCC displayed exceptional compatibility with the matrix and improved firmness with increasing concentration compared to control. The addition of CMC (>0.7%) led to the destabilization of the batter, resulting in its inability to form a cohesive protein network upon heating.	Texture	[105]
Mushroom (*Agaricus bisporus* and *Pleurotus ostreatus*) flours	2.5%, 5%	Beef patty	The addition of 2.5% mushroom flour from *Agaricus bisporus* and *Pleurotus ostreatus* enriched the fibre content and reduced the fact content by 25% and the salt content by 50%.	Salt and fat reduction/replacement	[81]
Sea spaghetti/konjac gel	3.3 g/100 g; 10.5 and 19.3 g/100 g	Frankfurtersausage	Konjac gel can be used to replace pork back-fat (reducing over 15% fat content) without significant changes in the sensory quality of frankfurters. The addition of a sea spaghetti/konjac gel (accompanied by reduction in salt) combination resulted in a more heterogeneous meat protein matrix with the seaweed integrated into the structure.	Salt and fat reduction/replacement	[85]
Plum powder and plum fibre marinade	0.06%	Chicken breast	The combination of plum powder and plum fibre marinade was found to have similar sensory and quality characteristics when compared to sodium tripoly-phosphate in boneless and skinless chicken breast fillets.	Phosphate reduction/replacement	[86]
Natural calcium powders fromegg and oyster	0.2%, 0.3%, 0.5%	Ground pork meat product	The combination of 0.2% oyster shell calcium and 0.3% eggshell calcium should enable the replacement of synthetic phosphate in the pork products with desirable properties.	Phosphate reduction/replacement	[106]
Cloves (*Syzygium aromaticum*) EO, Cinnamon (*Cinnamomum cassia*) EO	5% and 10%; 2.5% and 5%	Ground beef	The addition of 10% clove could completely inactivate *L. monocytogenes* in ground beef within 3 days post inoculation, irrespective of storage temperature. The addition of 5% cinnamon EOs could reduce 3.5–4.0 log CFU/g of *L. monocytogenes* after 7 days at 0 and 8 °C and after 60 days at −18 °C.	Antimicrobial functionality	[107]
Fresh plum juice concentrate (FP), dried plum juice concentrate (DP), spray dried plum powder (PP)	2.5%, 5%	Boneless ham	No significant differences in lipid oxidation among treatments. DP could contribute to a desirable red hue, therefore enhancing the colour and its stability in meat products.	Antioxidant functionality	[98]
Organic hydroxytyrosol (HXTo, 7% purity from olive tree leaves), synthetic hydroxytyrosol (HXTs, 99% purity), natural rosemary extracts (14.6% carnosic acid and 6% carnosol)	200 ppm	Lamb burger	HXT extracts and rosemary extract showed a good preservation activity, even higher than the control sample made with sulphites and synthetic antioxidants. The antioxidant activity (in vitro) of HXT extracts was found to be significantly higher than that of rosemary extracts.	Antioxidant functionality	[108]

#### Deep Dive: The Case of Meat Curing

Sodium or potassium nitrite salts are probably some of the most known and discussed additives in processed meat owing to their ability to stabilize cured meat characteristics and prevent the growth of *Clostridium botulinum*. The term “naturally cured” is another of those terms that is currently not well defined. According to the USDA, “uncured” meat products are those that are not allowed to contain purified (or synthetic) sources of nitrate or nitrite. All other ingredients, with the exception of those known for curing action or enhancement (sodium/potassium nitrate/nitrite, sodium erythorbate, sodium ascorbate, etc.) have no specific distinction, regardless of the nature of the product. Therefore, it is easy to understand the confusion that exists among consumers as products labelled uncured can have the same colour, aroma and flavour characteristics as traditionally cured products. Ingredients such as vegetable juice powders, have in fact, very high nitrate contents. Carrots can contain 117 ppm nitrate; celery, 27,000 ppm nitrate; beets, 2273 ppm nitrate; and spinach, 3227 ppm nitrate [109,110]. Celery juice converted to powder is one of the most compatible substrates with processed meat products, because it possesses very little vegetable pigment and only a mild flavour profile [110]. In one study, residual nitrate levels over time were observed to be higher in products containing powdered vegetable juice than those containing sodium nitrate. The researchers also found that there was no difference in colour and lipid oxidation between naturally-cured and conventionally-cured hams [111]. This observation is important in the context of commercial meat manufacture, since it demonstrates that natural products can be made with similar colour characteristics to conventionally-produced products. In another study by Choi, Bae and Jeong [112], authors successfully showed the potential of using kimchi powder along with powdered acerola juice as a substitute for synthetic nitrite in cured meat products, thereby providing options for consumers seeking “clean label products”.

Additionally, different ingredient companies have been working on the development of pre-converted vegetable juice powders (e.g., cherry powder), eliminating the requirement for the bacterial reduction step of nitrate into nitrite, thereby allowing for a faster processing time.

Most countries have stringent regulations in place regarding the use of sodium and potassium nitrate in cured meat products, despite its authorization for use [113]. From a European perspective, the current situation for consumer and manufacturer is less complicated, as nitrites added to food for technological functions (preservation or colouring) via other ingredients, such as vegetables, must still be listed as additives as per Regulation (EU) No. 1333/2008, thereby reducing confusion around labelling. The amount of nitrite added to meat products in Denmark is lower than that in other European Union countries, and this approach to reducing nitrite levels was approved by the Commission Decision (EU) 2018/702 [17]. Furthermore, a Food Chain Evaluation Consortium report indicates that since 2016, nitrite levels have been decreased in different meat products based on the product category and manufacturing procedure [17,114].

When it comes to natural antioxidant, the main challenge is to achieve similar effectiveness to synthetic antioxidants. Specifically, the most difficult part is to reach to incorporation levels for the compound to be effective while still maintaining the product quality characteristics, including flavour, aroma and colour. For example, in the case of natural curing replacers, it is necessary to standardize every ingredient batch against their nitrate/nitrite content, thereby requiring additional testing and cost. Moreover, specific natural antimicrobials, such as cultured celery powder, which is used to replace sodium nitrate and has proven effective against *Clostridium botulinum*, *Clostridium perfringens* and to a lesser degree *Listeria monocytogenes*, could cost 15–20-times more than its synthetic counterpart. However, as technology continues to develop, it is plausible to predict that natural antimicrobials will become more economically feasible to produce and utilise. This observation is of special safety concern when connected with phosphate reduction and that of other traditional water binders. Since less nitrite is present in the final product, expected shelf-life may also need to be adjusted in the absence of other substituting or supporting technologies. Alternatively-cured products are likely to have a shorter shelf-life than nitrite-cured products and care must be taken to ensure product safety [115]. Critically, it is necessary to target and understand those conditions that would support the outgrowth of *Clostridium botulinum* in processed meat systems.

## 3. “Clean Label” Processing

The existence of processed foods has been linked with human development for centuries, and this is not particularly surprising as the manufacture of processed foods has been linked to maximising the value of the food material produced and addressed human desires for greater choice and variation in the foods we consume. Yet, when consumers talk about “processed food”, they frequently refer to products that should be limited or avoided as they do not fit the concept of “natural” or “clean label”. Additionally, neophobia, disgust sensitivity and cultural values can also influence the perception of such technology [116]. As the “clean label” trend has matured, consumer expectations have also evolved. Therefore, the idea of a “clean label” processing approach as a means to replace or support ingredient modifications, improve product preservation and deliver some improvement around sustainability issues has also begun to grow in popularity. Processing techniques that may be perceived as “clean”, include cold-brewing, cold-pressing, fermentation and non–thermal technology methods, which give the perception of a product manufactured in a more natural way.

In this respect the technology with the most relevance to the meat industry is perhaps high pressure processing (HPP) as meat-dedicated units represent 21% of HPP machines installed globally [117]. The mechanism of HPP and its effects on meat quality have been recently and thoroughly described by Bolumar, Orlien [118]. By applying HPP treatments, it is possible to avoid thermal processing and better retain sensorial attributes, vitamins, antioxidants, and other compounds of added value. Furthermore, since there is no requirement to add preservatives, it is possible to obtain a “clean label” with a longer shelf-life. The many studies looking at the potential of HPP, pulsed electric field and ultrasound for salt and phosphate reduction have been reviewed by Pinton, dos Santos [119]. For cured meat products, the bactericidal effects of HPP reduces product salinity and the addition of antibacterial agents, resulting in more wholesome meat products that meet “clean label” requirements. In a recent study by Yang, Han [120], authors showed the potential use of HPP alone (200 MPa) to formulate reduced-fat and reduced-salt emulsion-type sausages without the need for any replacement. But it is not just “clean label” that is driving the increase in HPP uptake, its effects on texture and potential ingredients modifications is an area that is increasingly being explored. Sun and Holley [121] have shown that HPP treatment can influence meat protein conformation and induce protein denaturation, aggregation, or gelation, therefore playing a role in controlled texture modification. Moreover, the role of HPP as a tool to increase the saltiness of the product by modification in the meat structure, has also been explored [122]. The combination of HPP with processing hurdles, such as the inclusion of organic acids or salt replacers in processed meats, have been investigated by O’Neill et al. [123,124]. These authors showed that this approach could be a complete processing success as a hurdle strategy for extending both shelf-life and safety of low-salt meats, such as frankfurters, with a reduction in the salt content from 2.5 to 1.3% and cooked ham with a reduction in the salt content from 2.6 to 1.4 % [123]. The same authors showed that HPP could be used to accelerate marinade absorption into processed pork chops to increase product shelf-life, enhance sensory attributes and create niche value-added products [124]. One of the biggest challenges for “clean label” foods has been finding ways to keep food safe and attractive while retaining a reasonable shelf-life, without using artificial preservatives; these technologies are one of the key assets currently available for the industry to deliver such results. The widespread adoption of HPP technology will enable food manufacturers, and specifically meat processors, to penetrate new markets [125,126].

Challenge: With few exceptions, the use of technical methods in the food industry is not generally subject to labelling. However, country- or product-specific regulations or guidelines may necessitate labelling of treated products. The USDA Food Safety and Inspection Service approved HPP technology as a legitimate means for eliminating *Listeria monocytogenes* in processed meats [127]. In Europe, Novel Food Regulations and Food Information Regulations must be observed. Products which have not been manufactured with a common method are subject to the Novel Food Regulations, a process which may delay or increase the cost for market entry. Additionally, in the case of novel technologies, such as HPP, initial purchase cost and equipment set-up can range from USD 500,000 to USD 3 million, depending on specifications, thereby limiting applications [128].

## 4. Conclusions and Future Outlook

It is clear that what started as a retail idea or trend has now become a new norm. More than “clean label,” what the food industry is asked to address today is a clean image, which entails not only proving health and nutrition claims but also delivering societal and environmental benefit (e.g., packaging, sourcing, minimal use of natural resources). The “clean label” trend has opened up a number of exciting opportunities for the meat industry and can even offer possibilities to improve technological and sensory performance in plant-based meat alternatives and hybrid meat/plant products. To maximize its potential, product developers need to carefully source and use ingredients available to them when formulating their recipes, as the major risk of the “clean label” movement is the unnecessary removal of ingredients that have pivotal roles to play in terms of food preservation functions, raising concerns in term of health, food safety and shelf-life. It is vital for ingredients that enable consumer-friendly product labelling to continue to protect the consumer and adhere to local regulatory guidelines. Further research is required to more clearly understand the factors and boundaries that influence the perceptions of additives being “natural” as opposed to “synthetic” and the preferences relative to different food categories. Guidance on regulatory issues related to food ingredient usage and validation of ingredient/process effectiveness is currently missing, and whether an ingredient is considered “natural” or not is based on perception. With the rapid increase in popularity of plant-based and alternative proteins and the increasing number of consumer adopting a flexitarian lifestyle, a 33% drop in meat demand has been predicted by 2040 [129]. Therefore, it is crucial for the meat industry to continue working on new product development with a focus on convenience, cost, wellness and well-being benefits, aiming to attract consumers who want to try something new and to innovate in new spaces, such as hybrid. Consumer trust is key to a secure demand, therefore building transparency into the food chain remains a priority.

## Figures and Tables

**Table 1:** Example of clean label hydrocolloids and their functionality.

Hydrocolloid	Labelled as	Source	Key Functionality
Gums			
Acacia Gum	Gum Arabic	Tree Sap	Emulsifier
Agar		Seaweed	Gelation
Carboxymethylcellulose	Cellulose Gum	Cotton or Wood	Fibre
Carrageenan		Seaweed	Gelation
Cellulose Powder	Cellulose Gum	Cotton or Wood	Fibre
High-Acyl Gellan Gum	Gellan Gum	Fermentation	Gelation
Guar Gum		Seed	Viscosity/Fibre
High-Methoxyl Pectin	Pectin	Citrus or Apple	Gelation/Fibre
Inulin		Agave or Chicory	Fibre/Texture
Konjac Gum		Konjac Yam	Viscosity
Locust Bean Gum	Carob Gum	Seed	Viscosity
Sodium Alginate		Seaweed	Gelation
Tara Gum		Seed	Viscosity
Xanthan Gum		Fermentation	Viscosity
Native starches			
Potatoes	Potato Starch	Potatoes	Gelation
Maize	Maize Starch	Maize	Gelation/Viscosity
Barley	Barley Starch	Barley	Emulsifier
Wheat	Wheat Starch	Wheat	Gelation/Stabilizer
Tapioca	Tapioca Starch	Tapioca	Stabilizer
Proteins			
Pea	Pea Proteins	Pea	Emulsification/Foaming
Soy	Soy Proteins	Soy	Binding/Gelation
Gelatine	Hydrolysed Collagen	Animal Skin/Bones	Gelation/Foaming
Casein and Whey	Milk Proteins	Mammalian milk	Emulsification/Foaming
Ovalbumin	Egg Protein	Egg	Gelation/Foaming

Source: modified table from TIC Gums.

## Data Availability

Data is contained within the article.
